# Reuse of imputed data in microarray analysis increases imputation efficiency

**DOI:** 10.1186/1471-2105-5-160

**Published:** 2004-10-26

**Authors:** Ki-Yeol Kim, Byoung-Jin Kim, Gwan-Su Yi

**Affiliations:** 1 School of engineering, Information and Communications University, 103-6 Munji-dong, Yusung-gu, Daejon 305-714, South Korea

## Abstract

**Background:**

The imputation of missing values is necessary for the efficient use of DNA microarray data, because many clustering algorithms and some statistical analysis require a complete data set. A few imputation methods for DNA microarray data have been introduced, but the efficiency of the methods was low and the validity of imputed values in these methods had not been fully checked.

**Results:**

We developed a new cluster-based imputation method called sequential K-nearest neighbor (SKNN) method. This imputes the missing values sequentially from the gene having least missing values, and uses the imputed values for the later imputation. Although it uses the imputed values, the efficiency of this new method is greatly improved in its accuracy and computational complexity over the conventional KNN-based method and other methods based on maximum likelihood estimation. The performance of SKNN was in particular higher than other imputation methods for the data with high missing rates and large number of experiments.

Application of Expectation Maximization (EM) to the SKNN method improved the accuracy, but increased computational time proportional to the number of iterations. The Multiple Imputation (MI) method, which is well known but not applied previously to microarray data, showed a similarly high accuracy as the SKNN method, with slightly higher dependency on the types of data sets.

**Conclusions:**

Sequential reuse of imputed data in KNN-based imputation greatly increases the efficiency of imputation. The SKNN method should be practically useful to save the data of some microarray experiments which have high amounts of missing entries. The SKNN method generates reliable imputed values which can be used for further cluster-based analysis of microarray data.

## Background

DNA microarray is a popular high-throughput technology for the monitoring of thousands of gene expression levels simultaneously under different conditions [1]. The typical purposes of microarray studies are to identify similarly expressed genes under various cell conditions and associate the genes with cellular functions[2,3].

The analysis performed to meet the purposes of microarray studies mentioned above usually involves clustering genes according to their pattern of expression levels in various experimental conditions. In fact, cluster analysis means grouping samples (or genes) by similarity in expression patterns. To measure the similarity in cluster analysis, correlation distance and Euclidean distance are widely used[4]. Principal component analysis (PCA) is also a powerful technique when used with the clustering method to specify the number of clusters[5]. However, these widely-used methods in microarray data analysis can be both seriously biased and misled by missing values in the dataset[6-8].

Missing values of microarray data commonly occur during data preparation mainly due to imperfections in the various steps in DNA microarray experiments. One of the yeast microarray data sets shows that the number of genes having at least one missing value was 2419 of 6198 rows (genes) (in other words, 39 %)[9] and 566 of 918 rows (72.5%) [10]; and 1741 of 2364 rows (73.6%) [11] had missing values in other reports. As mentioned previously, some statistical analyses require complete data sets and one should discard the entire data in a row, usually all the values for one gene, that have a single missing value. The rows with missing values can be utilized for further analyses after the imputation of the missing values in many cases. Imputation has been used in many fields to fill the missing values in incomplete data using observed values. There are many different algorithms for imputation: hot deck imputation and mean imputation [7], regression imputation [12,13], cluster-based imputation [14], and tree-based imputation [15,16], maximum likelihood estimation (MLE)[17], and multiple imputations (MI)[17,18]. Proper selection of an algorithm for a given data set is important to achieve maximum accuracy of imputation.

Recently, several methods have been applied to the imputation of microarray data, including row average [7], singular value decomposition (SVD) [19] and KNN imputation [20] methods. In general, it seems the recently developed KNN-based method is most efficient. KNN imputation method is an improved hot deck imputation method [21] that uses the mean values of most similar genes for estimating missing values. The KNN imputation method can be considered a cluster-based method since missing values are imputed using selected similar genes. In the previously developed method, the efficiency of imputation was limited both in accuracy and computational complexity in that it did not efficiently use the information of the gene having missing values. The existence of missing values in a gene limits the use of other observed values of that gene in the conventional imputation method. In our work, this problem could be improved by using the imputed values sequentially for the later nearest neighbor calculation and imputation. We suggest a sequential KNN (SKNN) imputation method that boasts improved accuracy in estimation of missing values in a wide range of missing rates with high computational speed. We also suggest an EM-style sequential KNN (EM-SKNN) method that uses a sequential KNN method repeatedly to improve accuracy. We evaluated the efficiency of the SKNN imputation method through comparison with the known KNN-based method and other well known imputation methods such as maximum likelihood estimation (MLE) and multiple imputations (MI).

## Results

We evaluated the efficiency of our new SKNN method and the EM-SKNN method with three other imputation methods: KNN-based imputation, the MLE method, the MI method, by applying them to three different types of microarray data sets with different missing rates. The appropriate number (k) of nearest neighbors was dependent on the data types and missing rates. The RMS errors were minimal when *k *was 10 for time-series data and mixed type data regardless of the missing rate, and the RMS errors of the non-time series data showed similarly low values when *k *values were between 10 and 20. For comparison of different imputation methods, we used 10 for *k *which showed a minimal RMS error in every data type with different missing rates.

**Figure 1:**
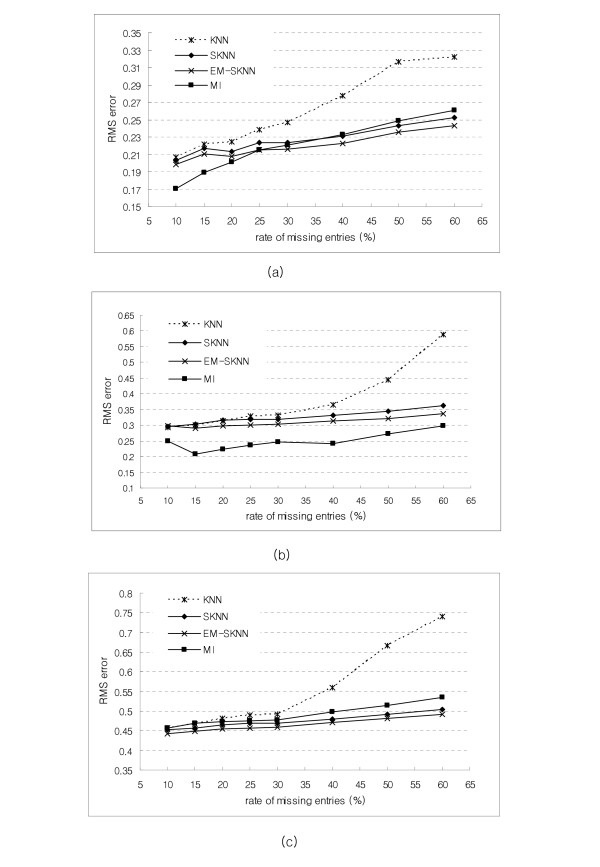
Comparison of the accuracy of KNN, sequential KNN, EM style sequential KNN, and MI methods for three different types of data sets. The accuracies were assessed by Root Mean Squared (RMS) error. Tested data sets were time series data set [22] (a), mixed (time series and non-time series) data set [23] (b), and non-time series data set [9] (c).

**Figure 2:**
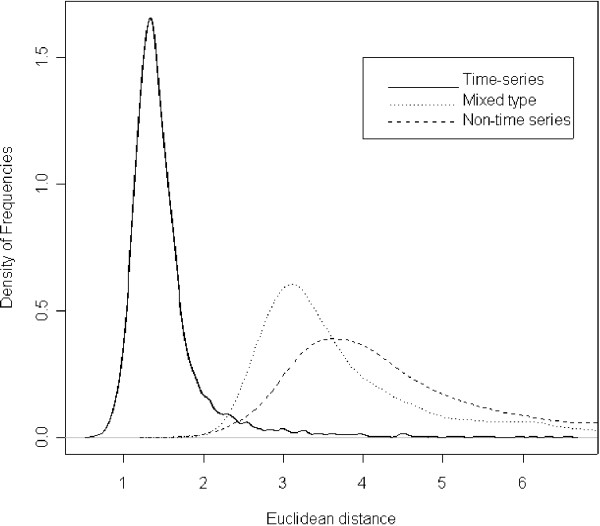
Comparison of distributions of the similarities between genes in different types of microarray data sets. The Euclidean distances were measured between a randomly selected gene and the rest of genes in each data set. The solid line, dotted line and dashed line represent for time series data, mixed data, and non-time series data respectively. The shape of the distribution implies the degree of similarities between genes in each data set.

The performance of the KNN-based imputation method depends on the similarity of *k*-nearest neighbors to be used for imputation. The overall similarity of the entire data set can affect, on average, the similarity of all possible *k*-nearest neighbors. The time-series data set, which has the narrowest distribution of Euclidean distances among genes, shows the least RMS error after imputation as we can see in Figure 1 and 2. Figure 1 also shows that the performances of the SKNN and EM-SKNN methods are better than that of the conventional KNN method over a whole range of tested missing rates. The range of RMS error by the new SKNN method, for example, was 0.194 to 0.269 in comparison with 0.194 to 0.324 of the KNN method in time-series data. The accuracies of our new methods are especially superior when the missing rate is over 30%. The RMS errors for time-series and non-time series data nearly approached their maximum values at missing rates of 50% and 60%, respectively, in the KNN method. The RMS error of the mixed data set is stable over a wide range of missing rates, but becomes unstable and increases dramatically after a 40% missing rate. The slight difference of KNN algorithm could lead to a large improvement in the accuracy of imputation at a high missing rate because the SKNN method is able to select more similar *k*-neighbors than the conventional KNN method as the missing rate grows. In the conventional KNN method, the selection pool and the dimension (or number of existing values for a gene) of the distance measurement of neighbor genes are reduced according to the increase of missing rate. In this situation, the method inevitably selects less related (or less similar) neighbors for imputation. In addition, the size of data set can limit the maximum missing rate for stable imputation. In our data sets, the size of a mixed data set is about 20% larger than other data sets, which may affect the mixed data set in terms of having stable RMS error in a relatively larger (10%) range of missing rate.

We tested the performance of other well known non-KNN-based methods such as maximum likelihood estimation (MLE) and multiple imputations (MI) methods. These methods are well known imputation methods but there has been no report on their application in microarray data analysis. The efficiency of the MLE method was much worse than the SKNN method for all tested data sets. The RMS errors in the MLE method were 0.11 to 0.33 in time-series data, 0.30 to 0.38 in mixed data, and 0.58 to 0.69 in non-time series data. The efficiency of the MI method was generally similar to SKNN but the former is more dependent on data types. The efficiency of the MI method was better at a lower missing rate, but slightly worse at a higher missing rate for the time-series data set. The MI method was worse than SKNN in terms of overall range of missing rate of non-time series data. However, the best imputation method for mixed data set proved to be the MI method. We can conclude that the MI method is as efficient as the SKNN method for the imputation of microarray data, even though the efficiency of the MI method experienced more fluctuations than the SKNN method depending on the data type.

The result is similar in a comparison of overall RMS error after imputation of a data set having unequally distributed missing entries over the columns. We show a comparison of one of the data sets (time series data set) in Table 1. As expected, the efficiency of the SKNN method is higher, especially for the data sets having a higher missing rate.

**Figure 3:**
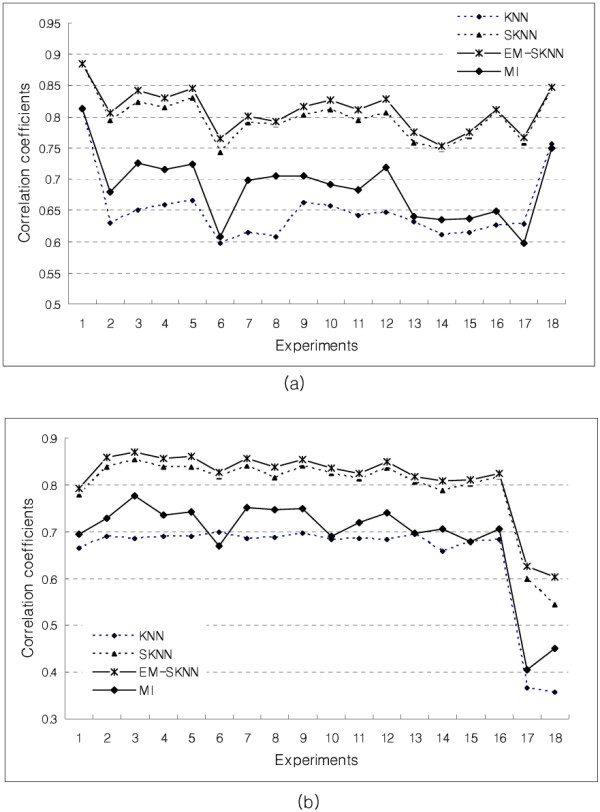
Comparison of the performance of KNN, SKNN, EM-SKNN, and MI methods by the correlation coefficients for each column between the original data and the imputed data of time-series data. The imputed data set was created by each imputation method from the data set having 50% of randomly (a) or non-randomly (b) generated missing entries.

For more careful estimation of imputation efficiency, we examined the structure of data after imputation. We calculated the Pearson correlation coefficients for each column (experiment) between original data and imputed data. The larger the correlation coefficient is, the better the relationship between original complete data and imputed data is preserved in a column. Figure 3 shows that the SKNN method preserves the structure of the original data set better than the conventional KNN method and MI method for all columns of the time-series data set. The situation was the same for the other data sets (data not shown). Interestingly, the MI method was much worse than the SKNN method, differing from RMS error analysis. This column-wise comparison gives us more specific information on the efficiency of imputation method. In Figure 3-b, we can see that the performance of SKNN is relatively better for the column with highly missing entries (column 17 and 18) than for other columns. Through measuring the means and standard deviations for each column of data sets, we discovered that the dispersion of values in a column does not affect the accuracy of KNN-based imputation.

The SKNN algorithm improves execution time for imputation. The computational complexities are approximately *O*(*m*^2^*n*^2^) in the conventional KNN method and *O*(*mn *log *m*) in the SKNN method for a matrix with *m *rows (genes) and *n *columns (experiments). This is because the sequential KNN algorithm imputes all missing values in a gene simultaneously with given nearest neighbors, while the conventional KNN method must calculate neighbors for each missing entry. The application of Expectation Maximization (EM) to the sequential KNN method marginally improved the accuracy in compensation for the increase of computational time proportional to the number of iterations. For MI methods, the execution time increased as M times of single imputation method when MI used M multiple imputation. Using the SKNN imputation method, it took 28.3 seconds on a Pentium IV 2.4 GHz computer to estimate missing values for a data set with 4489 genes, 18 experiments and a 40% missing rate. The processing time using the EM-SKNN method was proportional to the number of iterations.

## Discussion

The SKNN method offers better performance than the previously developed KNN method for both time series and non-time series microarray data sets and for data sets having different missing patterns. As the missing rate increased, sequential reuse of imputed data did not propagate errors of imputation as in the conventional KNN method. It showed the best improvement of accuracy for the data set with a high missing rate.

Notably, the SKNN method is also robust on the imputation of a data set with unequally distributed missing entries. A real microarray data set usually has non-random distribution of missing data. Furthermore, some systematic errors during the experiment can generate an abnormal increase in distribution of missing entries for the corresponding column of microarray data set. In this type of data, the SKNN method, which is especially efficient on the data set having heavy missing entries, can exert relatively more accurate imputation than other imputation methods as shown in our model data set. The MI method has not been well introduced in the field of microarray analysis, although it is a well known imputation method in other fields [18]. In comparison with the SKNN method, we discovered the potential of the MI method for microarray analysis. The MI method did not preserve original data structure as well as the SKNN method, but the overall RMS error was close to the SKNN method. The MI method is executed under the assumption of multiple normality of all dimensions of data. This assumption may not be satisfied in real-world data. Nevertheless, the performance of the MI method was much higher than the simple KNN method, which suggests that the MI method is practically applicable for the imputation of microarray data.

The computational complexity is reduced in the SKNN method for the dimension of both the number of genes and the experiments compared with the simple KNN method. Particularly, computation time can be saved substantially for microarray data with a large number of experiments. The SKNN method works efficiently in a wider range of missing rate with high speed. We want to emphasize that our results showed that the method using estimated values achieved even better accuracy than the method using only observed values in the case of the KNN-based imputation method. We suppose that this result could be applicable to other cluster-based analysis. It would be hardly acceptable for the experimentalist to use imputed data for further analysis. However, analysis could become more errorneous without imputation due to loss of information caused by missing values. The use of imputed data should definitely depend on the type of later process. If the next process is a cluster-based analysis, the genes with imputed values could be efficiently used, as we had good results for KNN-based imputation with the reuse of imputed values. For future works, it may be possible to integrate the imputation and gene clustering of microarray data for classification of genes with proper evaluation steps. This may offer more and better information sources of microarray data for the final decision of gene classification. All the procedures used in this paper are done by R-code and C++ and the programs are available upon request.

## Conclusions

The SKNN method is an especially efficient imputation method on data having high missing entries. It can be practically useful in saving data of some accidental microarray experiments having high missing entries. Our results also suggest that the imputed values generated by the SKNN method can be used reliably for further cluster-based analysis of microarray data.

## Methods

We developed and implemented SKNN and EM-SKNN methods for the imputation of microarray data, and we compared their accuracies with the previously developed KNN imputation method. Data sets used in this work were selected from publically available microarray data. The data sets were from a study of gene expression in yeast Saccharomyces cerevisiae cell-cycle regulation [22], calcineurin/crz1p signaling pathway [23], and certain environmental changes[9]. These data sets can be classified into time series data set [22], mixed (time-series and non-time series) data set[23] and non-time series data set[9]. The efficiencies of imputation methods were assessed by Root Mean Squared (RMS) error and correlation coefficients using three different data types as described later.

### KNN Imputation method

To assess the relative efficiency of the imputation methods, we implemented known KNN imputation method developed by Troyanskaya et al. (2001)[20]. The source code of the KNN imputation was available from the Helix group at Stanford University [24].

The matrix form of microarray data is composed of rows and columns that represent genes and experimental conditions respectively. Before any further process, the rows of original data sets containing missing values are removed to make complete matrices and test data sets for imputation methods were generated by random deletion of values in the complete matrices. The sizes of test data sets were 4489×18 for time series data set, 4380×24 for mixed data set, and 3779×22 for non-time series data set. The missing rates generated randomly in the test data sets were between 1% and 70% (1, 3, 5, 10, 20, 30, 40, 50, 60, 70). The occurrence of missing values can depend on the specific experiment in real miroarray data. Considering this case, we also generated test data set having missing values non-randomly along the columns which represent each experiment. The overall missing rate of data set was fixed to one of the value ranging from 10% to 60%. In a data set, the missing rates for two experiments (columns) were set to 80% and remaining columns have randomly generated missing entries.

In KNN method, *k*-nearest neighbor genes are taken from the whole matrix of the test data set except any genes that has missing value at the same position with the gene to be imputed. Euclidean distance is used as the metric to estimate the similarity of neighboring genes. To compare the similarity by this metric, each gene should have the same dimension and missing positions of values inside. Missing value is imputed with weighted average of the corresponding column of the *k*-nearest genes.

The weight of *i*^th ^gene is calculated as equation (1), where *k *is the number of selected genes and *D*_i _is the distance between *i*^th ^gene and a gene to be imputed.


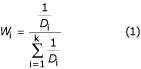


For the performance comparison of the imputation methods, we selected appropriate *k*-values for each data set and each method with different missing rates. Different *k*-values ranging from 1 to 500 were tested and we selected the *k*-values with the least error between imputed values and real values.

### Sequential KNN Imputation method

SKNN method that we suggest in this report is distinguished in two main points from previously implemented KNN method described above. In SKNN method, genes are ordered by its missing rate and the imputation was executed sequentially from the gene that had least missing rate. In addition, these sequentially imputed genes are used for the later imputation of the other genes. The test data set was separated into incomplete and complete set that has or has not missing values respectively. The genes in incomplete set were imputed by the order of missing rate. Missing value was filled by the weighted mean value of corresponding column of the nearest neighbor genes in complete set. Once all missing values in a gene are imputed, the imputed gene was moved into the complete set and used for the imputation of the rest of genes in incomplete set. In this process, all missing values in one gene can be imputed simultaneously from the selected neighbor genes in complete set. This reduced execution time from previously developed KNN method that should select nearest neighbors for each imputation.

### EM-style Sequential KNN Imputation method

EM-style imputation algorithm was originally suggested by Rich Caruana (2001)[25]. EM-style imputation is executed by two steps. It estimates missing values from observed values and improves accuracy of fill-in values through recursive process. We integrated EM-style imputation algorithm and SKNN method to increase the accuracy of imputation. All of missing values were estimated by SKNN imputation method at the first step. The estimated missing values were re-estimated by SKNN method again. In this second step, we could use newly imputed values to select *k*-nearest neighbors for the estimation of missing values. EM-style method executes this process repeatedly until the differences between newly updated values and previous values converge. Because all the imputed values were converged within less than 10 iterations, we did 10 iterations for the comparison of accuracy with the other methods.

### Maximum Likelihood Estimation (MLE) and Multiple Imputation (MI)

In MLE method, data set with missing values are centered, scaled, and sorted by the patterns of missing through the preliminary manipulations. Missing entries in a data matrix are estimated under the multivariate normal model with user-supplied parameters and observed data (non-missing entries in the data set). The parameters are estimated using imputed and observed data. A vector of parameters representing the MLE, means and variance-covariance matrix are returned by using EM algorithm. Missing values are estimated through this iterative process until estimated parameters converge. We executed MLE method to estimate missing values by using 'norm' library of R [26] based on the description of Rubin[17] for this work.

The whole MI procedure is made of three steps. They are imputation, analysis, and pooling processes. We applied only the first step, imputation process, of the three steps because our interest is to fill in missing values with estimated values. We used Predictive Mean Matching (PMM) as a method for missing values estimation. It uses a linear regression on observed variables to impute missing values. The estimated coefficients provide the mean vector and the variance matrix to generate multiple sets of coefficients that leads M imputed sets. M plausible values for missing observations were created by above MI algorithm and then the mean of M imputed values was filled in the missing value. We implemented Multiple imputation method using 'mice' library of R [26] based on the description by Rubin [17].

### Evaluation of imputation methods

The accuracy of imputation method was evaluated by calculating error between actual values and imputed values after missing values were estimated. The metric used to assess the accuracy of estimation was RMS error. RMS error was calculated as follows,


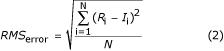


where *R*_i _is the real value, *I*_i _is the imputed value, and *N *is the number of missing values.

Besides RMS error, Pearson correlation coefficients were used to evaluate the sequential KNN method. Correlation coefficients were calculated between imputed data and complete data for each column. From this evaluation, we could find how the data structure of each column was preserved after imputation with different imputation methods.

## List of abbreviations

KNN: K Nearest Neighbor; SKNN: Sequential KNN; EM: Expectation Maximization; RMS: Root Mean Squared; PCA: Principal Component Analysis; MLE: Maximum Likelihood Estimation; MI: Multiple Imputation

## Authors' contributions

KK participated in the design of algorithms, performed statistical analysis and drafted the manuscript. BK participated in the design of algorithms and carried out C++ programming. GY conceived of the study, participated in its design and coordination, and finalized manuscript. All authors read and approved the final manuscript.

## Figures and Tables

**Table 1:** The RMS errors of different imputation methods for time-series data set having non-randomly generated missing entries.

missing rate(%)	KNN	SKNN	EM-SKNN	MI	MLE
10	0.2171	0.2247	0.2239	0.1573	0.2112
20	0.2233	0.2223	0.2175	0.2171	0.2602
30	0.2422	0.2285	0.2224	0.2277	0.2907
40	0.2778	0.2369	0.2288	0.2380	0.3079
50	0.3155	0.2450	0.2349	0.2495	0.3237
60	0.3210	0.2487	0.2476	0.2627	0.3381
